# On Some Aspects of Memory

**Published:** 1901-03-02

**Authors:** John Aikman


### On Some Aspects of Memory.

By John Aikman, M.D.

III.—ARCHITECTURE—(*Continued*).

So far physics lead us. But when a sound strikes your ear mechanism who can tell what particles are disturbed or what arrangement they take? Its detail is of the land of Chem; but the result makes up our memory.

We can decompose sunlight by the prism, and appraise the value of its different rays. There is a micro-organism named by Engelmann bacterium photometricum (Burdon Sanderson, *British Medical Journal*, September 16, 1893, p. 615), which illustrates the arrangement which freely moving cells make under the influence of light. If you place a drop of water containing this organism under the microscope and focus a beam of light on the smallest possible space of the field, the light spot acts as a trap, and the organisms crowd towards it, till the light spot attains a deep red colour, while the rest of the fluid becomes colourless.

Not only so, but if you decompose the light by a prism, these little rodlets show a preference for the spectral rays which are absorbed by their bodies rather than for those which pass through them.

A somewhat larger amæboid cell, the euglena viridis (*Clauss Zoology*, 1884, p. 195), will advance towards the blue line of the spectrum with an extemporised spindle-like process, and carry upon its point the red or orange colour of the other end of the spectrum.

This is practically the only evidence I can gather of polarisation in amæboid cells. The structure of the axon and dendron in the higher brain cells is that of bodies which should follow the law of polarisation.

By the law of polarisation, we mean the power of attracting opposites and repelling similars. Stated more simply—that which we have not attracts us, that which we already have fails to interest us. This law may rule the attraction and repulsion which causes movement of the elements in our nerve centres. It exists in nature, in light, and in electricity. It may any day be demonstrated in the physics of memory—not only in the making of it, but to show how we pass it into current coin.

We know, at least, that light arranges the colours of the foliage and flowers, that it bends trees towards the sun, and makes marked distinctions in the races of men. We wait for the demonstration of its effects upon the soft and tiny brain cell. Our eyes collect the light, and transmit its force to our brains with mathematical precision, and precision is the coin stamp of memory.

Pause for one moment and reflect upon how many of our brain cells die with us, without attaining even the first stage of development. Nature has often been called prodigal in its bounty. Among those cells which do develop there are varieties. There are simple memory paths, and there are cells which attain the position of excitors. The stellate cell fulfils the former condition. The distinction into axon and dendron seems to indicate the latter. More than that I cannot say.

IV.—LANDSCAPE AND PERSPECTIVE.

Life at its lowest means a power by which nutrition at least equals decay. Life in its higher grades means an excess of nutrition over decay with some surplus towards growth or activity.

The activity of muscle is measured by the heat produced. Let us consider how some such force would affect the development of our nerve cells. We believe that during life a force, or, as we call it, a current, is existent. The current passes by millions of pathways. A widely distributed force is little felt. The same force run through a narrow channel is recognised as a stream or torrent. A current of electricity run through a large wire imparts measurable heat. The same current run through a smaller wire generates obvious heat and light.

In sleep the dendrons of our higher nerve cells are in contact. During consciousness they are separate in whole or in part. In proportion to the limitation of their contact is our perception, our consciousness. So far as man can limit the contact of his dendrons he is on the alert, otherwise called "attentive."

This faculty of "attention" is man's "free will." It is the measure and the limit of it. It is that which he may yield to habits good and bad—habits which he follows without an appeal to his reason, without a call upon his attention.

The Rev. John Ward, who was Vicar of Stratford-upon-Avon soon after the time of Shakspere, puts into his diary this remark, "When man lost freewill woman found itt, and hath kept itt ever since" (page 111).

There is more than an apt joke in the quotation. We follow the direction of a woman more often from respect for herself than from an appeal to our reason. We sink our attention to a suggestion which we permit to find a ready path among our brain cells.

Habits are constructive when they add to our brain capital, and prepare the way for future work. They are destructive when they narcotise our free will and prevent due consideration. The habit of breathing is useful and permanent. We can suspend it of free will till our heart begins to miss beats. But we cannot do so without discomfort. We can acquire a trick of speech or manner till it becomes unconscious; and we may persevere in it until it becomes a nuisance to our neighbour, but no discomfort to ourselves. It requires a rude awakening and some effort of our memory to break that habit. But there is this difference—the useful habit cannot be broken without physical inconvenience. The useless habit can be broken by the tribunal of reason, when reason can rise to the occasion. A useless habit is often unreasoning imitation. And not only useless habits are broken by present need. A child has only learnt to walk. It falls ill, and when it recovers the power of walking is lost. The lost habit is useful, and is soon regained. The tribunal of reason has asserted its authority, and the habit is made permanent. Urgent need established the breathing act, so to speak, by one impression, and it is unassailable. Convenience, or culture, suggested the walking act, and it is only established after many repetitions.

Memory records are useful in such degree as they are clear, sharp, and permanent. A mere disturbance of the nutrition of the brain cells produces monstrosities of development and subsequent confusion. It falls short of a permanent imprint. Such indeed is the description of "worry." In its essence worry is an impression which leads to no conclusion. It fears rather than reaches the tribunal of judgment. The memory path is incomplete. There are gaps which only judgment can fill. There are cells whose faulty development dooms them to a perpetual winter. Worry has "only a torch for burning, no hammer for building." (Carlyle, "Sartor Resartus, Everlasting Yea")

(*To be continued.*)

